# From data to complex network control of airline flight delays

**DOI:** 10.1038/s41598-021-98112-7

**Published:** 2021-09-21

**Authors:** Xiang Niu, Chunheng Jiang, Jianxi Gao, Gyorgy Korniss, Boleslaw K. Szymanski

**Affiliations:** 1 Network Science and Technology Center, Rensselaer Polytechnic Institute (RPI), Troy, NY 12180 USA; 2 Department of Computer Science, Rensselaer Polytechnic Institute (RPI), Troy, NY 12180 USA; 3 Department of Physics, Applied Physics and Astronomy Rensselaer Polytechnic Institute (RPI), Troy, NY 12180 USA; 4 Społeczna Akademia Nauk, Łódź, Poland

**Keywords:** Mathematics and computing, Computer science, Statistical physics, thermodynamics and nonlinear dynamics, Complex networks

## Abstract

Many critical complex systems and networks are continuously monitored, creating vast volumes of data describing their dynamics. To understand and optimize their performance, we need to discover and formalize their dynamics to enable their control. Here, we introduce a multidisciplinary framework using network science and control theory to accomplish these goals. We demonstrate its use on a meaningful example of a complex network of U.S. domestic passenger airlines aiming to control flight delays. Using the real data on such delays, we build a flight delay network for each airline. Analyzing these networks, we uncover and formalize their dynamics. We use this formalization to design the optimal control for the flight delay networks. The results of applying this control to the ground truth data on flight delays demonstrate the low costs of the optimal control and significant reduction of delay times, while the costs of the delays unabated by control are high. Thus, the introduced here framework benefits the passengers, the airline companies and the airports.

## Introduction

Increasingly, dynamics of complex networks are defined by vast data sets collected about their activities via monitoring and sensing devices^1–4^. This creates a challenge for controlling such networks, since the control requires formal definition of network dynamics. We present a framework for deriving the formal definition of a network’s dynamics from data about its evolution. We show how to use this framework to provide a description of network failure dynamics^5^. The main contribution of this paper is to present application of this framework to a meaningful and complex example. Accordingly, we apply our new framework to the passenger airline networks of the United States. We focus on controlling one of the most common and frequent failures of these networks, which are flight delays.

Air traffic is an essential part of human mobility and global trade, but now it is outstripping the capacity and becoming frequently congested. Passenger airline flight delays and cancellations are prevalent and have socio-economic and environmental consequences^6–8^. Hence, these delays have caught the attention of decision makers and researchers from different fields^9,10^. To rein in the associated cost and increase the on-time performance of air traffic requires understanding of the causes of flight delays^11^. There have been extensive relevant studies^2,12–16^ that concentrate on addressing two core problems: how to model and how to mitigate the passenger airline flight delays. For example, Qin et al.^17^ proposed an agent-based model to describe the flight delay propagation mechanism, and applied the genetic algorithm to reschedule flights. Many approaches attempt to develop predictive models for flight delays, or for inferring flight delays causally. Hansen and Hsiao^18^ formulated an econometric model of the U.S. airline flight delays to account for direct causes, such as arrival queuing, weather conditions, and indirect ones, like seasonal influences and internal airline regulation and procedural changes. Among the vast body of approaches to predictive models of flight delays^14,19^, such as statistical analysis^3,4,11,18^, probabilistic models^20–22^, operational research^23,24^ and machine learning methods^2,25,26^, the network approaches based on complex network science^27^ have achieved much progress. Many techniques to model the mechanisms of flight delays employ direct acyclic graphs^28^, propagation trees^29^, Bayesian networks^20,22^ ot network-epidemic processes^30,31^. As for the mitigation strategies, with the exception of the traditional traffic management^32^, so far no effective and practical strategy has been found. There are some valuable discussions about renting spare aircraft to prevent and recover from schedule disruptions in^33,34^. The conclusions pointed to potentially prohibitive costs to airlines that may discourage them from adapting this approach to improve their on-time performance. In contrast, our analysis accounting for actual costs and past flight delays, shows that this aircraft renting may be economically viable in many airports and for many airlines.

Our framework involves the control of processes on networked systems^35–38^, which is well studied and achieves great success in many contexts, such as epidemic spreading^39,40^, information transmission^41^, and network resilience^37,42–44^. However, very few papers focus on mitigating delay propagation in airport networks. Among those with such focus the most relevant is Ref.^8^ whose approach models air traffic delay dynamics as topology transitions among a discrete modes of airport networks. Each such mode is associated with one characteristic airport topology. However, this approach has a limited capacity to deal with the complexity of air traffic networks. The airport networks built on such a scheme fail to recognize diverse interactions beyond pairs of airports, such as the interactions between pairs of flights, flights and airports. Another disadvantage is the expensive control of the mode transitions that usually require large infrastructure and technology investments. In contrast, our approach does not rely on any assumptions about the airport infrastructure. We build a multi-modal networked system over the flights and airports. It enables a flexible and effective control of the air traffic. Moreover, physical aspects of our control strategy are inexpensive, making it economical to apply.

Here we contribute a new solution by building a multidisciplinary framework using network science and control theory consisting of two components: (i) a model of dynamic of flight delays and (ii) a method for defining an optimal control strategy for this dynamic. The framework uses readily available ground truth data about flight delays currently collected by each airline to report it to the U.S. Department of Transportation’s Bureau of Transportation Statistics. We infer from this data the parameters for the model of flight dynamics. The proposed real time control strategy includes a *economic feasibility test* for renting a replacement aircraft in each airport. It requires that the cost of renting is lower than the cost of preventable delays. Our results demonstrate that this framework can effectively alleviate the delay propagation and significantly reduce the costs of delays to passengers, airlines and airports.

## Methods

### Flight delay networks

For each airline, we construct a flight delay network from historical data. It consists of graphs representing single aircraft flight delayed networks.

**Figure 1:**
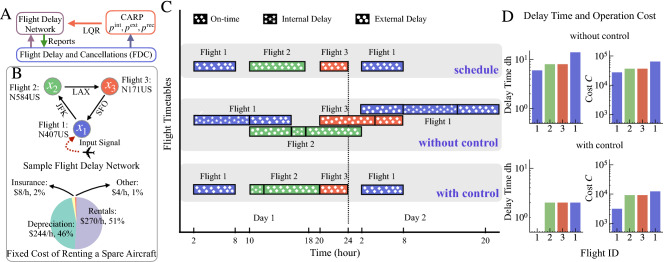
An illustration of the optimal control of a sample single aircraft flight delay network. (**A**) The flowchart of our framework for flight delay mitigation. The Flight Delays and Cancellations (FDC) data is the source for designing Flight Delay Network for each airline studied here. Using the CARP model, we formally define dynamics of the system, and its linearization and then apply the Linear Quadratic Regulator (LQR) to find optimal control. (**B**) A sample single aircraft flight delay network whose circular route includes three flights between three airports. Flight 1 N407US is selected as the driver node in controlling the flight delays. The pie shows the fixed cost of renting a spare twin-engine Boeing 737-401 to replace, if needed, the aircraft delayed in flight N407US from SFO to JFK airport. (**C**) The scheduled time of each individual flight, the actual flight time *with* and *without control*, under the assumption that some flights suffer delays. The details of these fights are discussed in the main text. (**D**) This part shows the delay time *dh* and cost *C* of each flight in the network produced by simulations with and without optimal control.

Figure 1A shows the flowchart of our framework for flight delay mitigation. The Flight Delays and Cancellations (FDC) data is the source for designing Flight Delay Network for each airline studied here and for the CARP model to estimate probabilities of model state transition, and costs of delays and aircraft rental. Using the CARP model, we formally define dynamics of the system, and its linearization and then apply the Linear Quadratic Regulator (LQR) to find optimal control. In the real time system, the FDC data will be available directly in real time from the airline running the system, simplifying processing.

Figure 1B shows a sample graph of a single aircraft flying a circular route consisting of three flights between three airports: the first six hour flight N407US flies from San Francisco International Airport (SFO) to John F Kennedy International Airport (JFK), the second eight hour flight N584US, from JFK to Los Angeles International Airport (LAX), and the closing loop third four hour flight N171US, from LAX to SFO. As usual for air transportation networks^45^, the flights are represented by nodes (each colored differently in the entire figure) and the airports by edges connecting a flight arriving into this airport to a flight departing later from this airport. The pie shows the fixed cost of renting a sample aircraft. This cost includes depreciation, rentals, insurance, and other costs (see Table 4-3 for detailed cost items and Table 4-6 for estimated costs in Ref.^46^).

Figure 1C shows the scheduled time of each individual flight, the actual flight time *with* and *without control*. The first panel shows flights without delays, while second and third panels with delays without and with control (see Supplementary Table S1 for detailed information about the internal, external and total delays and boarding, departure and arrival times of each flight). In this example, we assume that the first flight delays departure for 6 hrs because aircraft assigned to this flight undergoes an urgent maintenance procedure. This delay causes the internal delay for the first flight, and the external delays for the second and third flights (see Data Driven Dynamics in Methods for specific definition of internal and external delays). Moreover, the second flight is assumed to have 2 hrs of internal delay. As a result, it imposes 8 hrs of external delay on the third flight. Then the next day, the first flight suffers additional 6 hrs of internal delay and arrives in JFK 14 hrs behind the schedule. If the airline rents an aircraft for the first flight, it can go on time for passengers connecting with the second flight already in the airport, while the original aircraft is under maintenance. Therefore, a 6 hour internal delay for the first flight and three external delays of other flights will be avoided for all their passengers. Ultimately, four flights would be spared in total at 24 hrs of external delays and the next day flight 6 hrs of internal delay, so the total 30 hrs of delays. These and the internal and external delays are shown graphically in the figure.

Figure 1D compares the delay times, and the real costs of the three flights, with and without control. In our example, the difference between the costs of delays incurred without and with control amounts to 30 hrs, which costs much more than renting an airplane. Thus, the optimal control is beneficial in this case since eliminating the delay for passengers already in JFK is just 10% of the cost of control. In this example we assume large delays to illustrate cascading effects of the flight delays. In reality^47^, the average delay of a flight is about an hour (see Supplementary Fig. S1 for the real flight delay event samples over two days).

The flight nodes in single aircraft graphs of an airline are linked by connection edges. Such edge is drawn from a flight node $n_a(X)$ arriving at the airport *X* to a flight node $n_d(X)$ departing from airport *X* when flight $n_a(X)$ carries passengers connecting with flight $n_d(X)$. We use the average number of passengers connecting between such two flights as the weight of the drawn connecting edge.

The Flight Delays and Cancellations (**FDC**) dataset^47^ contains over five million flight records of 13 US passenger airlines (see Supplementary Table S2) active in the United States. Each record contains 31 properties of flights. We use nine of these properties shown in Supplementary Table S3 as node attributes in flight delay networks that we construct for each airline. These attributes are used to estimate parameters of our flight delay model.

### Data driven dynamics

Flight delay networks, like many real world networks, such as Internet and electrical power grids, exhibit cascading properties^5,48–50^. A single flight delay may cause cascading departure delays of many other flights as they wait at the gate for late-arriving connecting passengers. To describe and prevent such delay propagation of flights across the entire network, we introduce a **CARP** model that is based on an Markovian alternating renewal processes^5,51–55^. Let $x_i(t)$ be the continuous variable representing an expected state of flight node *i* at time *t* in the range [0, 1], where 1 denotes flight on-time and 0 means that it is delayed. The Markov property determines that the state of a node evolves according to the current state but is completely independent of the past states of the system. Specifically, CARP is formulated as follows:

1 $$\begin{aligned} {\varvec{x}}(t+1) = F({\varvec{x}}(t))+ G({\varvec{x}}(t),E)+B{\varvec{u}}(t)\triangleq {\mathcal {F}}({\varvec{x}}(t),E) + B{\varvec{u}}(t), \end{aligned}$$

where $F({\varvec{x}}(t))$ describes the endogenous delay dynamics on individual flights, $G({\varvec{x}}(t),E)$ defines the interactions among neighborhood flights through the underlying network *E* with *N* flight nodes, *B* is the *input matrix* that identifies usually a small set of *driver nodes* through which the input signals ${\varvec{u}}(t)$ are entered to drive the system to a desired state. Often selection of the nodes that belong to a driver node set is difficult as different choices impose different costs of control^56^. In our application, any airport in which an aircraft of a needed size is available for timely replacement of the delayed plane can make a driver from a node representing a flight with the delayed arrival in this airport. In our simulations, we assume that the number of passengers waiting for the delayed plane is a historical average. In reality, the airline may make a decision about providing a plane for delay control dependent on a sufficient number of passengers waiting for the delayed plane. Such real-time dynamic decisions will improve benefits of our method compared to our simulations using just historical averages.

The nonlinear functions $F({\varvec{x}}(t))$ and $G({\varvec{x}}(t),E)$ can vary for different applications. For flight delays, both are defined as parametric functions $F({\varvec{x}}(t)) = {\varvec{p}}^{int}\circ [1-{\varvec{x}}(t)] + {\varvec{p}}^{con}\circ {\varvec{x}}(t)$ and $G({\varvec{x}}(t),E) = [E^T {\varvec{x}}(t)] \circ {\varvec{p}}^{ext}\circ [1-{\varvec{x}}(t)]$, where $\circ$ denotes the Hadamard product.

The model relies on three probabilities ${\varvec{p}}^{int}$, ${\varvec{p}}^{ext}$ and ${\varvec{p}}^{con}$, where $p_i^{int}$ and $p_{i}^{ext}$ are the probabilities of the flight represented by node *i* being delayed by an internal or external factor, and $p_{i}^{con}$ is the probability that this flight retrains its state. Since the causes of each flight delay are recorded in FDC, we estimate the probability of each cause directly from the data. Thus, we equate $p_i^{int}$ and $p_i^{ext}$ to the frequencies with which flight *i* was delayed in the data for internal and external causes, respectively. Denoting by $f_i^{rec}$ the frequency of the flight *i* being on time after experiencing a delay computed from the data, we estimate $p_i^{con}=1-f_i^{rec}$.

To estimate the needed probabilities, we made some assumptions to simplify processing the records in FDC. The most important are (i) we consider each record as a single event, related to others only by connection edges, that are either an on-time or a delayed flight, regardless how long the delay is; (ii) we assign the same time interval between the arrival of the previous flight and the departure of the next flight for all flights and airlines, and after this interval expired all flights depart simultaneously; and (iii) we merge the records of all flights between the same departure and arrival airports regardless of the aircraft actually used by each airline.

From FDC, we create a flight delay network for an airline including the associated adjacency matrix of connection edges *E* based on the scheduled arrivals and departures of all flights of an airline in a given airport. With the data driven dynamics CARP, we are able to simulate all flights in the network and the resulting dynamics of direct and cascading flight delays. At the initial state ${{\varvec{x}}}(0)$, we randomly assign to each flight delayed status based on the probabilities estimated for this flight, otherwise the on-time status is awarded to this flight. If no control is exercised, i.e. ${\varvec{u}}(t)=0$, the network will automatically evolve to an equilibrium state ${\varvec{x}}_s$, such that ${\varvec{x}}_s=F({\varvec{x}}_s) + G({\varvec{x}}_s,E)$. The state ${\varvec{x}}_s$ is probably undesired, and may correspond to widespread presence of flight delays.

To limit the impact of flight delays, we need to develop a well-defined control strategy, watchfully monitoring and manipulating the input signal ${\varvec{u}}(t)$ to steer the system away from ${\varvec{x}}(0)$ to an ideal inactive state ${\varvec{x}}_f={\varvec{0}}$, where no flight delay happens, at the cost acceptable to airlines. There is a problem, however, since unlike many existing control strategies for complex systems that require ${\varvec{x}}_f$ to be an attractor^57,58^, ${\varvec{x}}_f$ usually is not an attractor of the flight delay system. Thus, we seek a compromise solution and control the system to a desired state with the minimum cost.

### Optimal Control of Flight Delay Networks

The optimal control is an optimization problem in search of an optimal sequence of input signals $\{{\varvec{u}}(0)$, ${\varvec{u}}(1)$, $\ldots$, ${\varvec{u}}(\tau )\}$ to minimize a function $J({\varvec{u}},{\varvec{x}}; \tau )$ regarding all involved states ${\varvec{x}}$ and input signals ${\varvec{u}}$ within $\tau$ time steps’ evolution.

#### Linearization

 Generally, nonlinear dynamics are extremely hard to solve directly for optimal control. A common practice is adopting the linearization technique^59^ to reformulate the nonlinear dynamics in advance, and then to solve an approximated linear system instead. As described in Eq. (1), the governing dynamics of flight delay networks are nonlinear, so we linearize them at an *operating point* $(\bar{{\varvec{x}}}, \bar{{\varvec{u}}})$ for optimal control. Let’s denote the reformulated linear system as follows:

2 $$\begin{aligned} \tilde{{\varvec{x}}}(t+1)\approx A\tilde{{\varvec{x}}}(t)+B\tilde{{\varvec{u}}}(t), \end{aligned}$$

where $\tilde{{\varvec{x}}}(t) = {{\varvec{x}}}(t) - \bar{{\varvec{x}}}$, $\tilde{{\varvec{u}}}(t) = {{\varvec{u}}}(t) - \bar{{\varvec{u}}}$ and $A=\partial {\mathcal {F}}/\partial {{\varvec{x}}}_{|{\varvec{x}}={\varvec{x}}_s}$. There are a variety of choices of $(\bar{{\varvec{x}}}, \bar{{\varvec{u}}})$, but some may ease the solving of the input signals ${\varvec{u}}(t)$ for optimal control, e.g., the *equilibrium* ${\varvec{x}}_s$ *of the system without control* that satisfies $\dot{{\varvec{x}}}_{s}={{\varvec{0}}}$ and ${\varvec{u}}_s={\varvec{0}}$. Let $\bar{{\varvec{x}}}={\varvec{x}}_s$ and $\bar{{\varvec{u}}}={\varvec{u}}_s$. Once $\tilde{{\varvec{x}}}$ and $\tilde{{\varvec{u}}}$ for optimal control are solved, it is straightforward to derive the real-time control strategy: ${\varvec{x}}(t)=\tilde{{\varvec{x}}}(t) + {\varvec{x}}_s$ and ${\varvec{u}}(t)=\tilde{{\varvec{u}}}(t)+{\varvec{u}}_s$, $\forall t\le \tau$.

#### Linear quadratic regulator

LQR is an optimal control operating on a linear system like Eq. (2), widely adopted in many science and engineering applications^59,60^. It minimzes a quadratic function of the state vector ${{\varvec{x}}}$ and the control input ${{\varvec{u}}}$:

3 $$\begin{aligned} J({\varvec{x}},{\varvec{u}};\tau ) = J_f({\varvec{x}}(\tau );Q) + \sum _{t=0}^{\tau -1} J_t({\varvec{x}}(t),{\varvec{u}}(t); Q,R), \end{aligned}$$

where the matrices *Q* and *R* are design parameters to penalize ${\varvec{x}}$ and ${\varvec{u}}$, respectively; $J_f({\varvec{x}}(\tau );Q)={{\varvec{x}}}^T(\tau )Q{{\varvec{x}}}(\tau )$ is the cost at the final time step $t=\tau$, while $J_t({\varvec{x}}(t),{\varvec{u}}(t); Q,R)={{\varvec{x}}}^T(t)Q{{\varvec{x}}}(t)+{{\varvec{u}}}^T(t)R{{\varvec{u}}}(t)$ is the stage cost at $t<\tau$. Because *J* is quadratic, an optimal control sequence is uniquely determined as the solution of an induced backward Riccati recursion equation^59^.

The entries in *Q* and *R* are weights applied to $${\varvec{x}}$$ and $${\varvec{u}}$$, respectively. Many applications require an analytic formulation of the optimal feedback control law, therefore *Q* and *R* need to be symmetric semi-positive and definite^60^. In this paper, we consider them as diagonal matrices, i.e. $$Q=diag(q_1^2,q_2^2,\ldots ,q_N^2)$$ and $$R=diag(r_1^2,r_2^2,\ldots ,r_N^2)$$, where $$q_i$$ is the average delay cost to a passenger, while $$r_i$$ is the rental cost of a replacement aircraft. According to the statistics provided by the Federal Aviation Administration, the average loss caused by a flight delay is approximately $49 per hour per passenger^61^. There were 9.5 million domestic flights carrying about 895.5 million passengers in 2015^62^. On average, each flight services 94 passengers. Thus, the average delay cost for each flight is $$c_d=49\times 94=\$4,606$$ per delay-hour. Moreover, with the average delay time $${ dh}_i$$ in hrs for each flight *i* recovered from FDC, we can estimate $$q_i = c_d\times dh_i$$.

The input signals ${\varvec{u}}$ in the optimal control may be of various forms, depending on the scenarios. In our framework, we proposed adding a backup airplane to an airport on the route as an input signal. According to the operating and fixed costs of renting an aircraft presented in Tables 4-6 in Ref.^46^, the average rent cost of an aircraft is $c_r=\$526$ per usage hour. From FDC, we retrieve the average flight time ${ fh}_i$ of each flight *i*, and estimate $r_i=c_r\times { fh}_i$.

#### Total operating cost

The quadratic objective function *J* in Eq. (3) for optimal control guarantees the uniqueness of the solution, but its physical implication is indefinite. To have a quantitative perspective of how much the optimal control can reduce flight delays and how the states ${\varvec{x}}$ and the input signals ${\varvec{u}}$ affect such performance, we formulate a linear function

4 $$\begin{aligned} C({\varvec{x}}, {\varvec{u}}; {\varvec{q}},{\varvec{r}})=\sum _{t=0}^\tau [{\varvec{q}}^T {\varvec{x}}(t) + {\varvec{r}}^T {\varvec{u}}(t)] \end{aligned}$$

for the total operating cost of enforce controls over a flight delay network. This cost has two sources: the expected delay cost and the aircraft renting cost, where ${\varvec{q}}=(q_1,q_2,\ldots ,q_N)^T$ and ${\varvec{r}}=(r_1,r_2,\ldots ,r_N)^T$ are weights assigned to ${\varvec{x}}$ and ${\varvec{u}}$. The total operating cost *C*, alternatively called *total cost* or *cost* in short, is used in our analysis to quantify the performance of the optimal control or no-control (i.e., ${\varvec{u}}(t)=0$, $\forall t\le \tau$) strategies in mitigation of flight delays.

## Results

### Optimal control for the sample flight delay network

We apply an optimal control approach to a small sample flight delay network to show how it reduces the flight delays with minimal cost defined by Eq. (3). We use the estimated flight delay cost matrix *Q* and the aircraft renting cost matrix *R* based on real data (see Fig. 1A and Methods).

**Figure 2:**
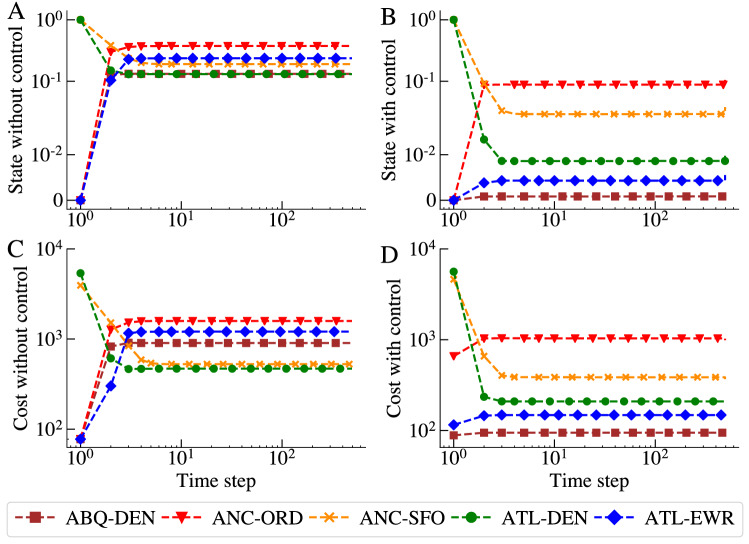
State and cost trajectories of five representative flights by United Airlines. (**A, C**) The state and cost trajectories of flights without control. (**B, D**) The state and cost trajectories of flights under LQR control.

Figure 2 shows a realistic example of the state and cost trajectories of five representative flights operated by the United Airlines (UA). Figure 2A,C show results for the system without control while those in B and D were simulated with control on. In all cases, a stable state is reached in a few steps. Figure 2A,B compare the probabilities of delays for all flights. Without control, these probabilities range from 0.1 to 0.8, while with control they decreased a little for two flights but a lot for the remaining three flights. Figure 2C,D show costs. Compared to costs in C, the costs with control on decreased a bit for the two flights but dropped significantly for the remaining three flights. This realistic example created with real data confirms the potential for strong improvements of both the average delay probability and the average costs of flights with control compared to those without it. However, it is likely that the two flights with the modest reduction of costs and probabilities will occasionally fail the feasibility test when the number of passengers awaiting for connection in the airport is below average.

### Optimal control for the real airline flight delay networks

**Figure 3:**
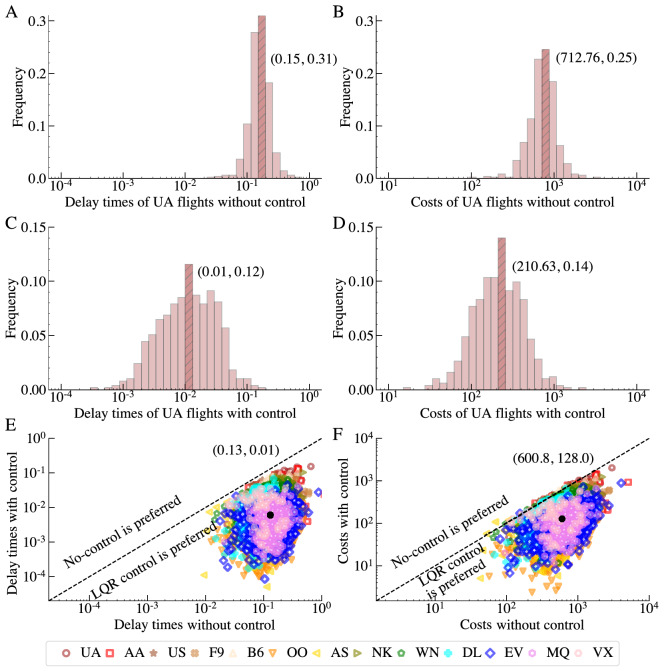
Comparison of flights with or without control in terms of the delay time *dh* and the total cost *C* . (**A, B**) The distributions of the delay time *dh* and the total cost *C* of flights on the United Airlines (UA) without control. (**C, D**) The distributions of the corresponding *dh*’s and *C*’s with the optimal control. The striped bars indicate the bins where the median values of *dh*’s or *C*’s fall, and the numbers in parentheses are these median values and their associated frequencies. The median *dh* with control (**C**) is less than 10% of that without control (**A**), while the associated total cost with control (**D**) is only about 32% of that without control (**B**). (**E, F**) The difference between with and without control for flights from all 13 airlines in terms of *dh* and *C*, respectively. Each individual shape represents one flight, and the shape form and color indicate which airline operates it. Black dots show the median values of the corresponding *dh*’s or *C*’s. On average, LQR reduces the delay time by over 92%, and reduces the total cost by around 80% relative to the no-control strategy. To make the comparisons consistent, all experiments are ran for a fixed number of steps, i.e., $\tau =N$, which equals to the size of the flight delay network, but value of $\tau$ negligibly affects the results.

 With the data-driven dynamics CARP from FDC, we evolve the system without any control and with the LQR based optimal control for $\tau =N$ time steps. Figure 3 compares the results that demonstrate that LQR can be adopted to successfully control the flights of all 13 US domestic passenger airlines with a minimum cost. For each airline, both the overall delay time and the cost of flights are dramatically reduced with LQR. First, Fig. 3A,B show histograms of the delay time and the total cost without control in the logarithmic horizontal scale for a flight on the United Airlines. Figure 3C,D display these histograms with the optimal control. The median delay time with control (C) is less than 10% of that without control (A), but the ranges of distributions of delays and costs vary strongly among flights. Each of the UA flights saves at least 50% of the cost. Fig. 3E,F show the ranges of distributions of delays for all airlines. Each dot indicates the costs incurred from LQR or with no control for an individual flight. Most flights fall below the anti-diagonal line, with only a few outliers on or above the line. On average, the reduction of the cost is about 75% and of the delay time 90%. Yet, we notice that for some airlines, such as US Airways (US), the delay times and costs are relatively similar among all flights; while for other airlines, such as American Eagle Airlines (MQ), some flights arrive close to the scheduled time, while others experience lengthy delays. Overall, the ranges of distributions of delays vary strongly among airlines.

**Figure 4:**
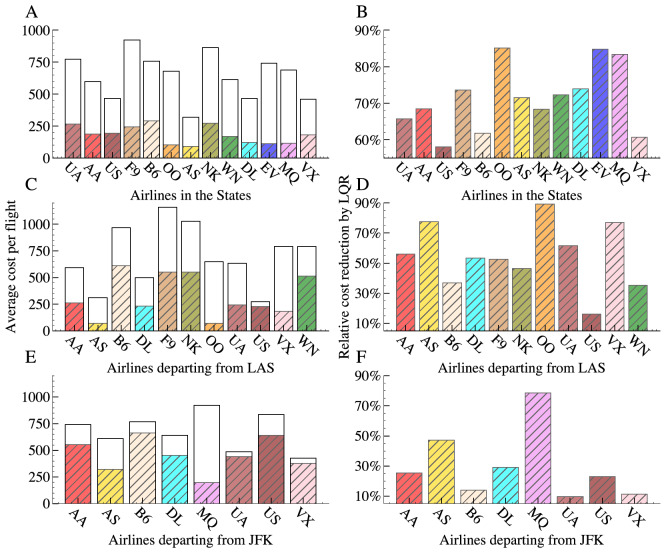
Reduction in delay cost by the optimal control LQR on airlines in the United States and airlines departing from two airports LAS and JFK. (**A, C, E**) The total cost per flight *with* and *without* control for each airline. The shaded areas represent costs with control and the blank areas represent costs saved by LQR. (**B, D, F**) The relative percentage of the saved costs by LQR.

According to Fig. 4A,B, almost every single flight benefits from the optimal control, and the flights of each airline save at least 50% in cost. In addition to the study of individual flights, we also evaluate the impact of optimal control of airports. Figure 4C–F show the costs with or without control for two airports LAS and JFK (see Supplementary Fig. S2 for the costs with or without control for 316 airports in the United States, Figs. S3 and  S4 for the top 10 costs of flights and airports for each airline). For each airline, we average the cost of an airport over all departing flights. The optimal control significantly reduces the costs of most of the airports. However, the reductions are the largest for the airports in the central areas of the U.S. In contrast, the airports in big cities or coastal areas achieve more moderate cost reductions because of their relatively high passenger flow and heavy air traffic. Figure 4A,B explicitly show the costs of two of the most representative airports: LAS on the west coast and JFK on the east coast, each of which serves more than eight airlines. Under LQR control, the cost of each airline in both airports reduces. In LAS, SkyWest Airlines (OO) benefits most, while in JFK the biggest beneficiary of control is American Eagle Airlines (MQ).

## Discussion

Flight delays are a widespread and highly visible problem in the passenger aviation industries. They often bring uncertainties into air travel and cause financial losses to all involved parties, including the passengers, the airlines, the airports, and even the insurance companies.

We developed a generic data-driven framework for an optimal control of networked systems that focuses on system failures^5,53,56,63^. Here, we apply this framework to the flight delay data collected in 2015. We discuss how to formalize dynamics of flight delay propagation using historical data and how to define the optimal control strategy to minimize the flight delay at the reasonable costs. To this end, based on historical data, we create a flight delay network for each of the 13 US passenger airlines. Then, we estimate the optimal parameters for a model of nonlinear dynamics of the flight delay propagation mechanism. Using this model and the LQR approach we define the optimal control over the flight delay networks. The results from simulations of the solution demonstrate that the framework effectively suppresses the flight delay propagation and significantly reduces the costs of delays. These simulations are run under a pessimistic assumption that flights have an average number of passengers. In real time use of this approach, the system can compute the exact cost of renting a replacement plane to the next flight of the delayed airplane and compare it with the cost of delay for the actual number of passengers on the delayed plane or in the airport waiting to board the arriving plane for a connecting flight. Hence, the replacement will only be made if it is economically viable according to the actual data. This further validates the applicability of this framework to different scenarios, such as airline flight delays demonstrated here, the global economy risks discussed in Ref.^5^, and COVID-19 government strategies for slowing pandemics presented in Ref.^56^.

To summarize the novelty of our approach. The key to our approach is constructing a flight delay network that enables us to precisely predict the consequences of not controlling the delay (letting the delay happen unabated) versus renting (or using an airline own) airplane to control delay and then implement that choice that is less costly. Without flight delay network, comparing such costs was impossible. This network also enables us to predict the expected number of delays from past performance and to establish the expected minimum and maximum demands for rented airplanes and their costs.

The data needed to create the networks are currently gathered by each airline by request from the US government. None of the previously proposed approaches used this precious data to build a flight delay network and compute quickly costs of unabated and abetted failure crucial for our approach.

In short, our approach maps a flight delay problem into a standard model of a complex machine in which each flight is a part of the machine that processes elements (passengers) into final products (passengers arriving in their destinations or canceled trips). Each part (flight) interacts with other parts (flights connecting with the original flight), and each part (flight) has as crucial parameters its probability of delay, number of passengers and each passenger itinerary. Thus, the problem becomes the standard machine efficiency optimization to which we apply tools of control theory. Thus, we creatively use the existing data and a system control theory to solve the problem that on a first glance is far from the standard model of machine failure control.

As suggested in^45^, the method can be enhanced by merging flight delay networks of airlines cooperating within airline alliances. In future work, we will address some related issues to make the framework broadly applicable. The most challenging extension is to design a direct solver for a model with non-linear dynamics. We expect that it might be more costly computationally, but the optimal control will be more efficient. The most interesting extension is the control of coordinated delays.

Currently, our model only considers delays of individual planes that are fairly independent from each other. However, in case of storms, security breaches, or personnel strikes, the entire airports may be affected and departures and arrivals may be suspended for hrs at a time. Real-time models deployed by airline will be able to use real-time precise data about flights and their schedules, the delay costs and aircraft renting to accurately predict the cost and effectiveness of using additional planes, improving benefits over those estimated here.

## Supplementary information

Supplementary Information.
