# Supplementary material for: From data to complex network control of airline flight delays
Source: Sci Rep. 2021 Sep 21;11:18715. doi: 10.1038/s41598-021-98112-7 (PMC8455581; doi:10.1038/s41598-021-98112-7)
Supplement: Supplementary file 1 — Supplementary Information. [file 41598_2021_98112_MOESM1_ESM.pdf]

Supplementary Material:
From Data to Complex Network Control of Airline Flight Delays

Xiang Niu[1,2], Chunheng Jiang[1,2], Jianxi Gao[1,2], Gyorgy Korniss[1,3], Boleslaw K. Szymanski[1,2,4*]
[1]Network Science and Technology Center, Rensselaer Polytechnic Institute (RPI), Troy, NY 12180, USA
[2]Department of Computer Science, Rensselaer Polytechnic Institute (RPI), Troy, NY 12180, USA
[3]Department of Physics, Rensselaer Polytechnic Institute (RPI), Troy, NY 12180, USA
[4]Społeczna Akademia Nauk, Łódź, Poland
*boleslaw.szymanski@gmail.com

Supplementary Tables

Table S1. Timings of the sample flight from Figure 1D without delays and with delays without and with control.

Flight	Time					
	External	Internal	Boarding	Departure	Arrival	Total Delay
	**No Delay**					
1	0	0	2	2	8	0
2	0	0	2	10	18	0
3	0	0	2	20	0 (next day)	0
1	0	0	2	2 (next day)	8 (next day)	0
	**Delay without Control**					
1	0	6	2	8	14	6
2	6	2	2	18	2 (next day)	8
3	8	0	2	4 (next day)	8 (next day)	8
1	8	6	2	16 (next day)	22 (next day)	14
	**Delay with Control**					
1	0	0	2	2	8	0
2	0	2	2	12	20	2
3	2	0	2	22	2 (next day)	2
1	2	0	2	4 (next day)	10 (next day)	2

Table S2. List of 13 passenger airlines and their IATA codes.

IATA Code	Airline	IATA Code	Airline
AA	American Airlines Inc.	NK	Spirit Airlines
AS	Alaska Airlines Inc.	OO	Skywest Airlines Inc.
B6	JetBlue Airways	UA	United Airlines Inc.
DL	Delta Airlines Inc.	US	US Airways Inc.
EV	Atlantic Southeast Airlines	VX	Virgin America
F9	Frontier Airlines Inc.	WN	Southwest Airlines Co.
MQ	American Eagle Airlines Inc.		

Table S3. Nine features extracted from the flight records in FDC for construction of flight delay networks.

Feature Name	Description	Feature Name	Description
Airline	airline identifier	Late Aircraft Delay	delay caused by the aircraft
Airline Delay	delay caused by the airline	Origin Airport	departure airport
Arrival Time	actual arrival time	Scheduled Departure	planned departure time
Departure Time	actual departure time	Tail Number	aircraft identifier
Destination Airport	arrival airport		

Supplementary Figures

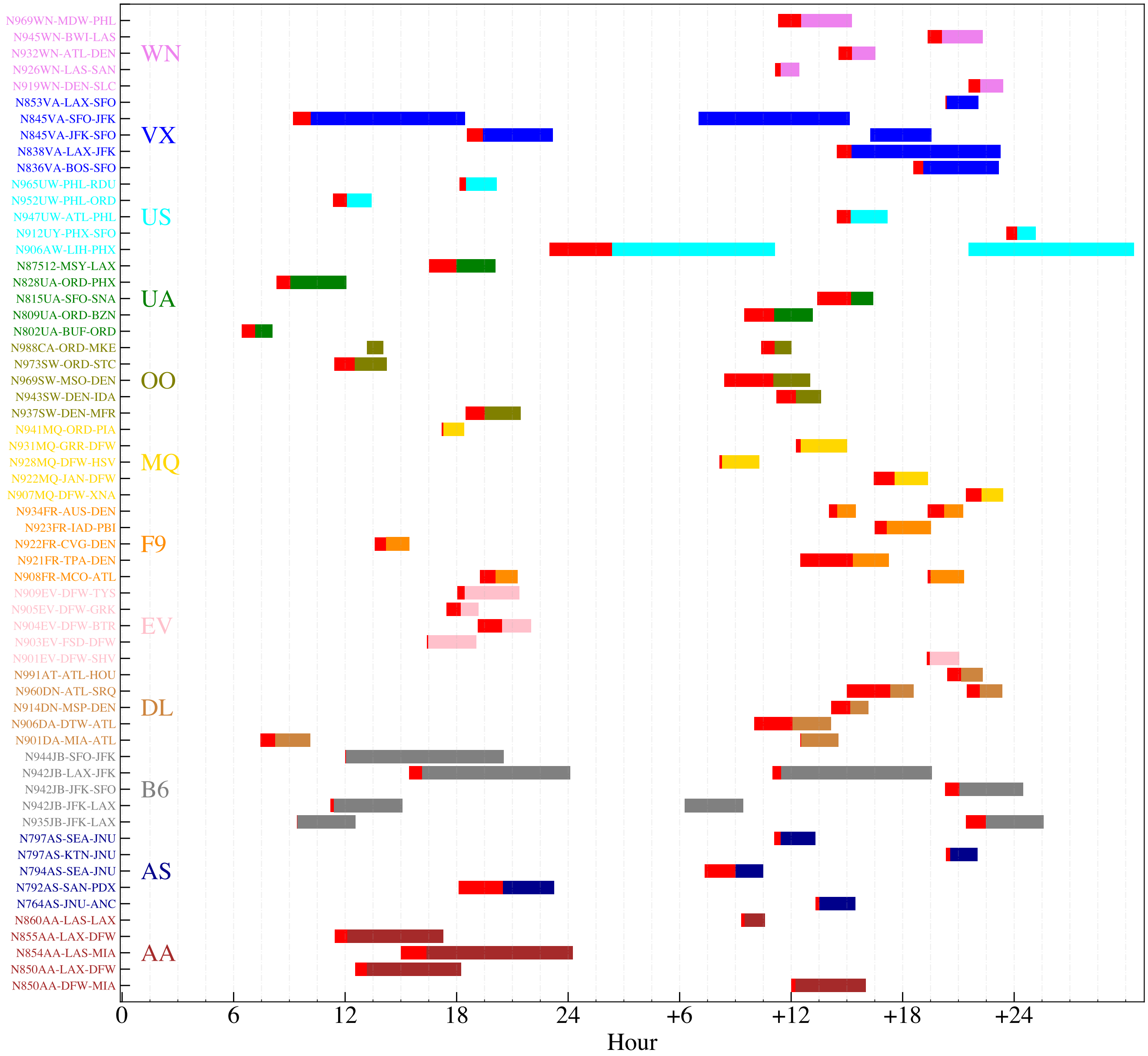

Figure S1. Representative routes with two-day flight delay from January 1st to January 2nd, 2015. Among the 13 studied airlines, most have two-day delay except Spirit Airlines (NK). For each airline, five representative flights are selected for illustration. Each event is labeled by the flight identity that is composed of the tail number, the departure airport and the arrival airport.

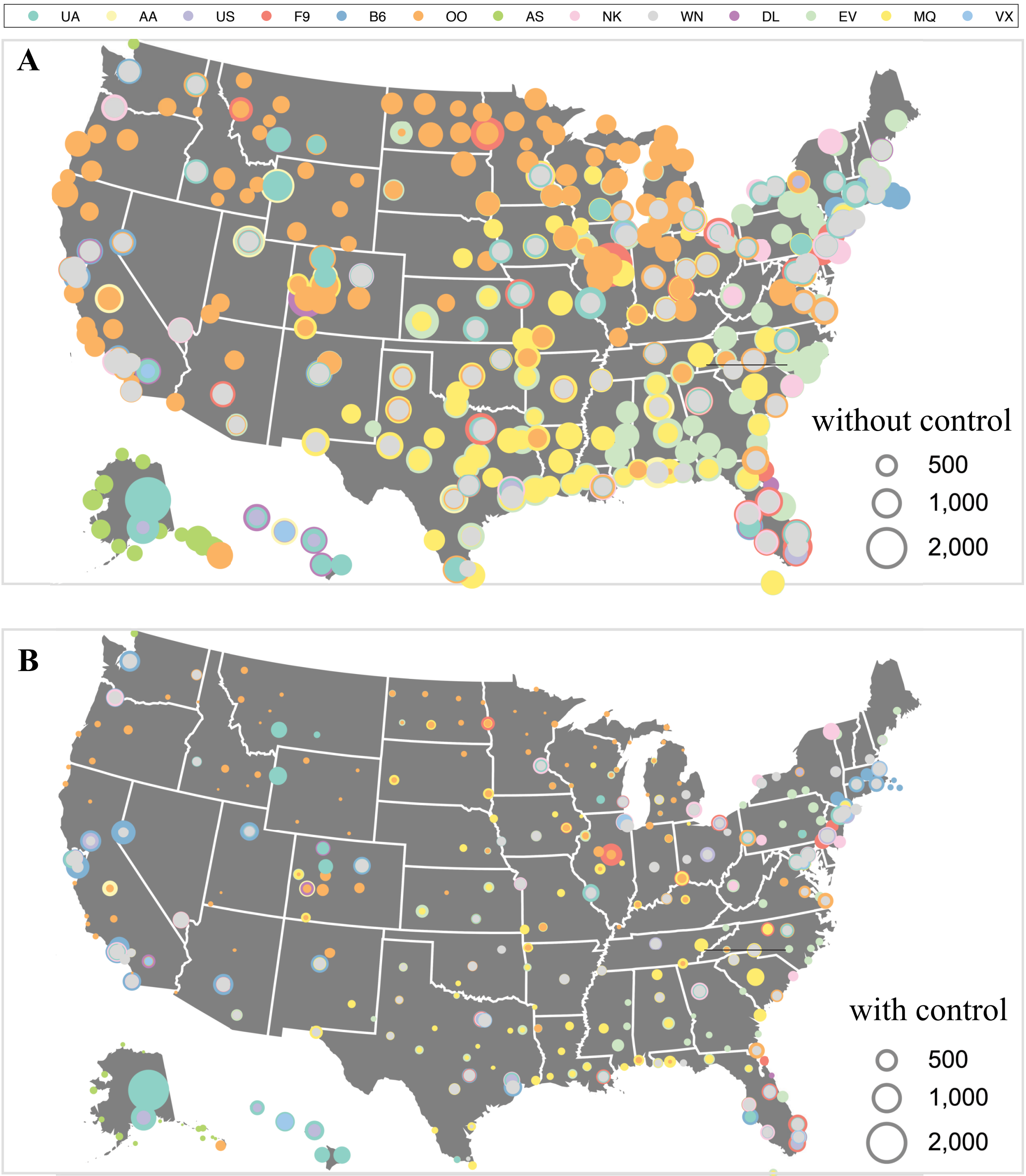

Figure S2. Reduction of the delay cost by the optimal control of airports in the United States. The cost of flight delays *without* (**A**) and *with* (**B**) control of departure airports in the United States. Each color represents a distinct airline with the same mapping of colors to airlines as in Fig. S1. Size of each node is proportional to the average cost of flights departing from the airport represented by this node. Comparing visually the sizes of pairs of nodes without and with control in the two maps, it is clear that most of the airports enjoy meaningful reduction of costs of delays.

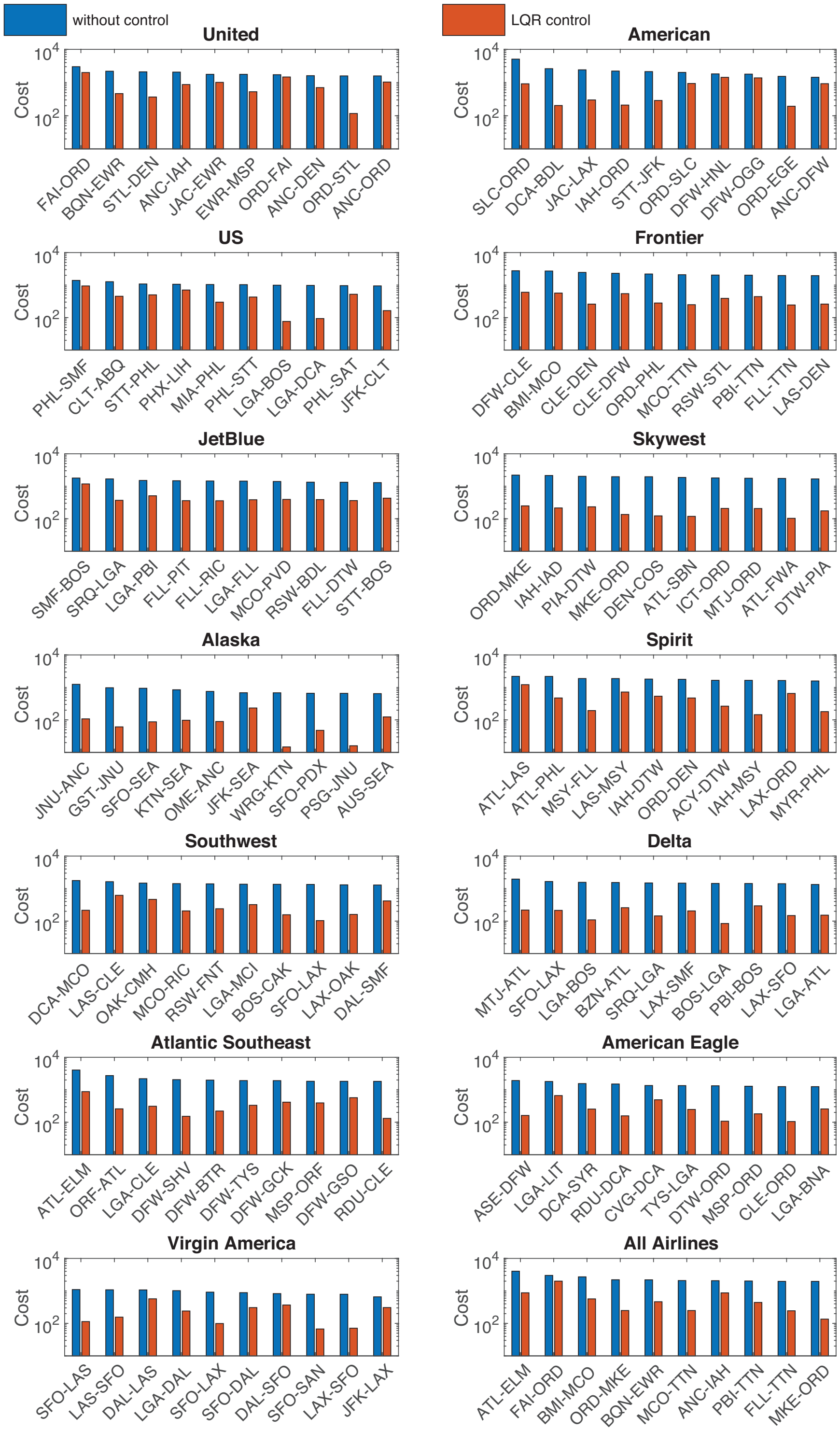

Figure S3. **Top ten most costly flights in terms of delay risk.** Except the *All Airlines* panel, each panel describes the cost distribution over all flights by one airline under LQR control (in blue) or without control (in red). The *All Airlines* panel demonstrates the average cost distribution of top 10 most costly flights by all 13 studied airlines.

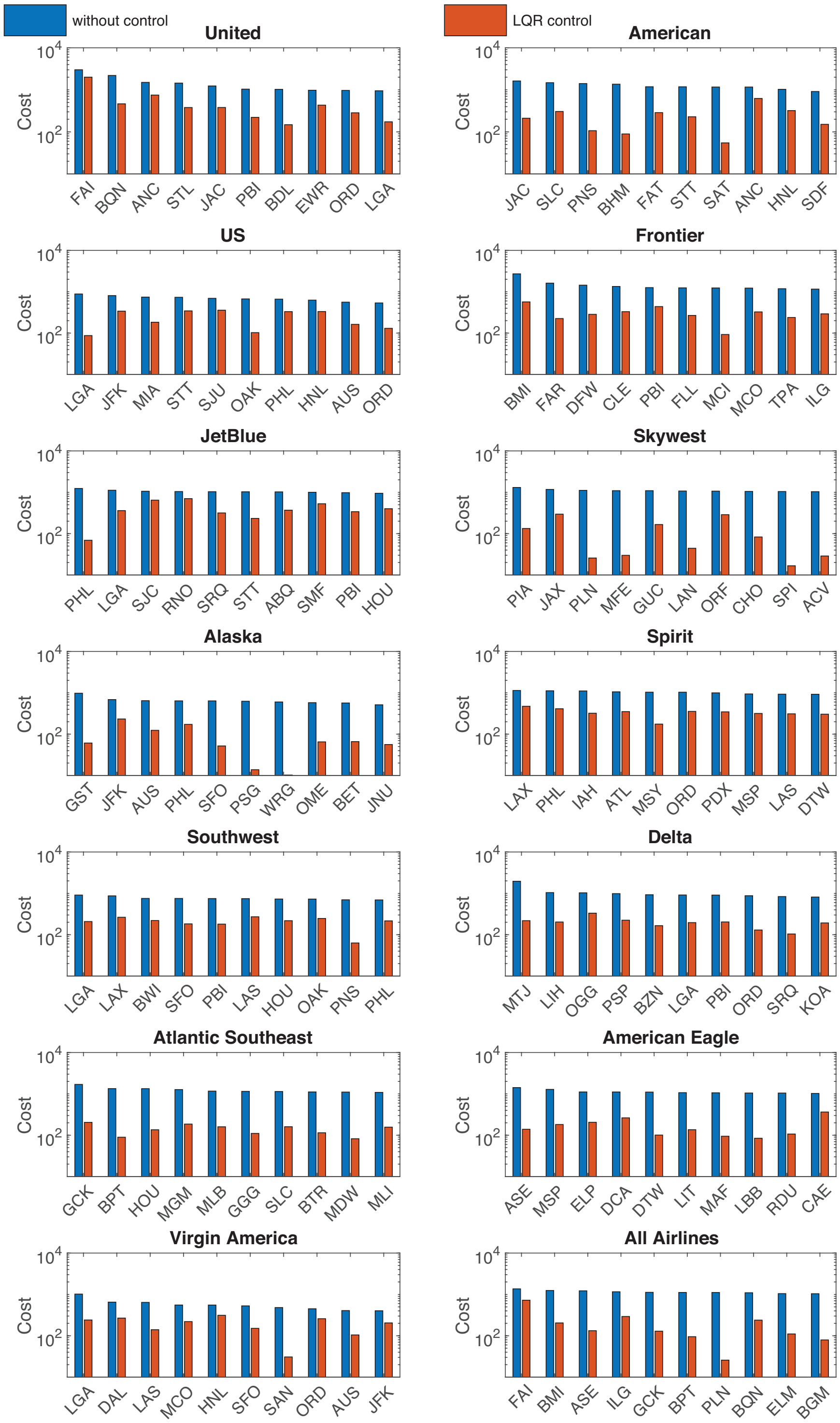

Figure S4. Top ten most costly departure airports in terms of delay risk. Except the *All Airlines* panel, each panel describes the cost distribution over the departure airports of flights by one airline under LQR control (in blue) or without control (in red). The *All Airlines* panel demonstrates the average cost distribution of top 10 most costly departure airports by all 13 studied airlines.
